# Ensemble neural network models for stability prediction and optimization of hydraulic structures considering uplift pressure and exit gradient

**DOI:** 10.1038/s41598-025-25149-3

**Published:** 2026-01-09

**Authors:** Elsayed Elkamhawy, Mohamed S. Sawah, Mohammed Tawfik, A. S. Ismail

**Affiliations:** 1 Faculty of Engineering, Zagazig University, Zagazig, 44519 Egypt; 2 Department of Computer Science, Faculty of Information Technology, Ajloun National University, P.O.43, Ajloun, 26810 Jordan; 3 Department of Information Systems, Al-Alson Higher Institute, Cairo, Egypt; 4 Department of Cyber Security, Faculty of Information Technology, Ajloun National University, P.O.43, Ajloun, 26810 Jordan; 5 Faculty of Science, Zagazig University, Zagazig, 44519 Egypt

**Keywords:** Optimization algorithms, Hydraulic structure stability, Uplift pressure mitigation, Piping, Exit gradient, Engineering, Mathematics and computing

## Abstract

This study aims to develop a novel ensemble modeling approach that integrates artificial neural networks with finite element analysis to optimize the stability of hydraulic structures, particularly through the design of cutoff wall configurations. The research investigates the effects of varying cutoff wall positions and inclination angles on key parameters such as uplift pressure, seepage discharge, and exit gradient. Numerical simulations were performed using Geostudio SEEP/W to analyze seepage patterns across multiple configurations. The proposed methodology combines a Feed-Forward Neural Network (FFNN), XGBoost Regressor, and Support Vector Machine (SVM) with a Genetic Algorithm (GA) to create a predictive optimization framework. The findings reveal that the optimal cutoff wall inclination angle for minimizing both uplift pressure and exit gradient is 165° across all positions, while for seepage discharge, the optimal angle varies by position, ranging from 60° to 120° and increasing incrementally by 15° from upstream to downstream. The ensemble model demonstrated robust predictive performance across 5-fold cross-validation trials, achieving mean R-squared values of 0.99 ± 0.01 for uplift pressure, 0.94 ± 0.02 for seepage discharge, and 0.97 ± 0.01 for exit gradient. The small standard deviations indicate consistent performance across different data partitions, validating model stability and generalizability. The Genetic Algorithm results closely aligned with the numerical model outputs, validating the robustness of the proposed framework. This study introduces a significant improvement over traditional analytical methods by providing an integrated approach that enhances the safety and efficiency of hydraulic infrastructure design, particularly under complex conditions where conventional techniques may fall short.

## Introduction

Hydraulic and diversion structures, including dams, weirs, and levees, are essential elements of water resource management systems. Despite their importance, they are often subjected to significant challenges such as seepage, piping, and uplift pressure. These issues can compromise the stability and functionality of the structures, potentially leading to failures that result in substantial economic and social consequences. To address these challenges, engineers employ various mitigation strategies, including the implementation of cutoff walls and various drainage systems^1–4^. Seepage is a common issue in hydraulic structures built on permeable foundations, arising from the difference in water levels between the upstream and downstream sides. The effects of seepage can be classified into three main categories: (1) uplift force, which reduces the shear resistance between the structure and its foundation and lowering the safety factor against sliding and overturning; (2) seepage discharge, which affects the overall water balance; and (3) exit gradient, a critical parameter in designing hydraulic structures to prevent the piping phenomenon^5^. Piping occurs when the exit gradient exceeds a critical threshold, leading to soil erosion and potential structural failure. Recent work by Rath et al.^6^ applied explainable machine learning (XGBoost with SHAP analysis) to predict exit gradients in hydraulic structures, highlighting anisotropy and cutoff wall depth as dominant factors. Excessive uplift pressure and the piping phenomenon are recognized as the leading causes of hydraulic structure failures^7^. To address these risks, it is essential to reduce both the net uplift pressure and the exit gradient, as these measures are critical for ensuring the safety and stability of hydraulic structures. One effective solution is the implementation of cutoff walls at the foundation sides of these structures^2^. Cutoff walls are impermeable barriers typically constructed using materials such as steel sheet piles, concrete trenches, grout curtains, or impervious blankets. Their strategic placement helps reduce seepage discharge and exit gradients, thereby providing protection against uplift pressure and preventing piping^1,2,8,9^. In addition to cutoff walls, comprehensive drainage systems are widely used to control seepage flow and mitigate its adverse effects. These systems often include drainage galleries, drainage tunnels, and draining wells, which work collectively to manage water pressure and flow beneath hydraulic structures^2,10^. By integrating these measures, it can be significantly enhanced the durability and safety of hydraulic infrastructure, reducing the risk of failure and its associated economic and social impacts.

Seepage beneath hydraulic structures has been extensively studied, leading to the development of several theories, including those by Bligh^11^, Lane^12^, and Khosla and Bose^13^. Bligh’s creep length theory^11^ describes the path of seepage flow under hydraulic structures, assuming a linear uplift pressure distribution and proportional energy loss along the creep path. Lane^12^ introduced the weighted creep theory, assigning different coefficients to horizontal (0.33) and vertical (1.0) seepage paths to account for variations in flow resistance. Khosla and Bose^13^ proposed a more advanced approach, modeling seepage flow using concentric ellipses and hyperbolas to calculate uplift pressure distribution. While Khosla’s theory is generally more reliable than Bligh’s and Lane’s, it becomes complex and less accurate for anisotropic foundations or complicated geometries^5^. Analytical solutions based on the Laplace equation can provide precise uplift pressure distributions, but are often impractical for complex engineering scenarios due to their mathematical complexity^14^. To address these limitations, numerical methods, particularly the finite element approach, have gained widespread use. These methods divide the foundation into finite elements, enabling accurate calculations of uplift pressure, seepage flow, and exit gradient, even for complex boundary conditions^8,15,16^.

In recent years, significant research efforts have been directed toward analyzing uplift pressure, seepage flow, and exit gradient using numerical approaches, particularly the finite element method. These studies have investigated the influence of varying cutoff wall positions and inclination angles under static conditions^1,2,4,5,9^. One notable advancement is the integration of finite element modeling with genetic algorithms (GA) to determine the optimal placement and inclination of cutoff walls in hydraulic structures, especially for specific cutoff depth-to-floor length ratios under static conditions^17–20^. Furthermore, research conducted by Javanmard et al.^21^ has examined the interaction between the core and foundation of hydraulic structures equipped with cutoff walls, considering both static and dynamic loading conditions. Haghdoost et al.^22^ used finite element modeling to investigate how penetration depth, position, and inclination of internal cutoff walls influence seepage and hydraulic gradients in homogeneous earth dams. While Huang et al.^23^ examined seepage patterns in core rockfill dams with double cutoff walls in deep overburden, providing practical insights for complex geological conditions.

Artificial neural networks (ANNs) are widely employed in civil and environmental engineering to assess the consistency between measured and predicted values of key parameters^24–27^. The effectiveness of ANNs in modeling stems from two key advantages: first, they enable theoretical analysis, and second, they provide a reliable framework for predicting output parameters based on comparable input data. When studying the hydraulic performance of cutoff walls beneath hydraulic structures under static conditions, ANN modeling serves as an efficient alternative for data analysis, delivering quick and accurate results with relatively low complexity^28–30^. Additionally, solutions based on the radial basis function (RBF) method have also demonstrated satisfactory accuracy^31^. A recent study of Shakouri et al.^32^ combined numerical modeling with advanced soft computing methods to improve prediction accuracy for earth dam performance.

As highlighted in previous literature, there is a lack of optimal solutions for determining the ideal cutoff wall position and inclination angles for hydraulic structures. While ANNs and ensemble modeling approaches have been widely employed for predictive analysis, these methods often focus on correlating input and output parameters without providing actionable design optimization strategies. This study aims to bridge this gap by introducing an innovative approach that combines finite element techniques with GA. Unlike existing neural network or ensemble modeling approaches, which primarily emphasize prediction accuracy, the proposed method integrates numerical modeling with optimization algorithms to predict and optimize key design parameters simultaneously. Specifically, this research develops a coupled finite element-GA model capable of determining the optimal location and inclination angles of cutoff walls beneath hydraulic structures, considering complex boundary conditions and anisotropic foundation properties. This dual capability, precise hydraulic behavior modeling, and design optimization represent a significant advancement over traditional predictive methods. By providing a comprehensive solution for improving the design and stability of hydraulic structures, this study contributes to developing safer and more efficient water resource management systems, ultimately protecting lives, property, and infrastructure in surrounding areas.

## Materials and methods

### Governing equations

Seepage flow through porous media is governed by Darcy’s law and Poisson’s equation (Eqs. 1 and 2, respectively). Water flows from regions of higher total head to lower total head due to the influence of the hydraulic gradient.

1 $$\:q=-kA\frac{\partial\:h}{\partial\:l}$$

2 $$\:{\frac{\partial\:}{\partial\:\text{x}}(K}_{x}\frac{\partial\:\text{h}}{\partial\:\text{x}})+\frac{\partial\:}{\partial\:\text{y}}\left({K}_{y}\frac{\partial\:\text{h}}{\partial\:\text{y}}\right)+Q=\frac{\partial\:{\uptheta\:}}{\partial\:t}$$

In this context; $\:q$ represents the seepage discharge $\:({m}^{3}/s/m)$, $\:k$ represents the hydraulic conductivity coefficient $\:(m/s)$, $\:A$ is the area of the cross section $\:\left({m}^{2}\right)$, $\:\partial\:h/\partial\:l$ is the hydraulic gradient, $\:{K}_{x}$ and $\:{K}_{y}$ are hydraulic conductivity in horizontal and vertical directions, respectively $\:(m/s)$, $\:\text{h}$ is the total water head $\:\left(m\right)$, $\:Q$ is the applied boundary flux, $\:{\uptheta\:}$ is the volumetric water content, and t is time.

### Numerical simulation

In this study, the Geo-Studio (2019) software, specifically the Seep/w module^33^, was employed to evaluate key parameters such as uplift pressure, exit gradient, and seepage flow. Seep/w is a finite element-based program specifically designed to model and analyze water seepage and pressure distribution in porous media. It is capable of handling both steady-state and transient flow conditions within a two-dimensional plane strain domain, making it a versatile tool for solving a wide range of geotechnical and hydraulic engineering problems. For the analysis, the foundation soil was assumed to exhibit isotropic and homogeneous properties, with a saturated hydraulic conductivity of $\:{10}^{-5}\:m/s$. This simplification allowed for a consistent and reliable representation of the soil’s behavior. A steady-state seepage analysis was conducted for all configurations of the cutoff wall systems. The cutoff wall was modeled as an impermeable boundary, effectively preventing water flow through it, which provided valuable insights into the seepage patterns and pressure distribution within the foundation. Cutoff-wall penetration depth, as highlighted in Shakouri^34^, plays a critical role in controlling seepage and hydraulic gradients. Although penetration depth was held constant in this study to isolate the effects of wall position and inclination, it remains a key factor for future optimization scenarios. Additionally, the numerical simulations assumed a fully saturated condition for all materials within the hydraulic structure foundation model. This assumption was essential to accurately capture the interaction between water and soil particles, ensuring realistic and reliable results for the seepage analysis. Furthermore, current reviews of EL-Molla, D.A., and Kilit, M^35^. have synthesized seepage control, detection, and treatment strategies in embankment dams, including optimized designs of drains, cores, and seepage barriers, as well as modern monitoring and diagnostic tools. The numerical machine learning framework presented in this study is compatible with such practices and can be extended to incorporate these elements in future work. By considering these conditions, the study aimed to thoroughly investigate the seepage behavior, uplift pressures, and exit gradients in the presence of cutoff walls under all scenarios (i.e., different locations and inclination angles). The use of Seep/w enabled a detailed and comprehensive analysis of the hydraulic structure foundation, contributing to a deeper understanding of its performance under the specified conditions.

**Fig. 1:**
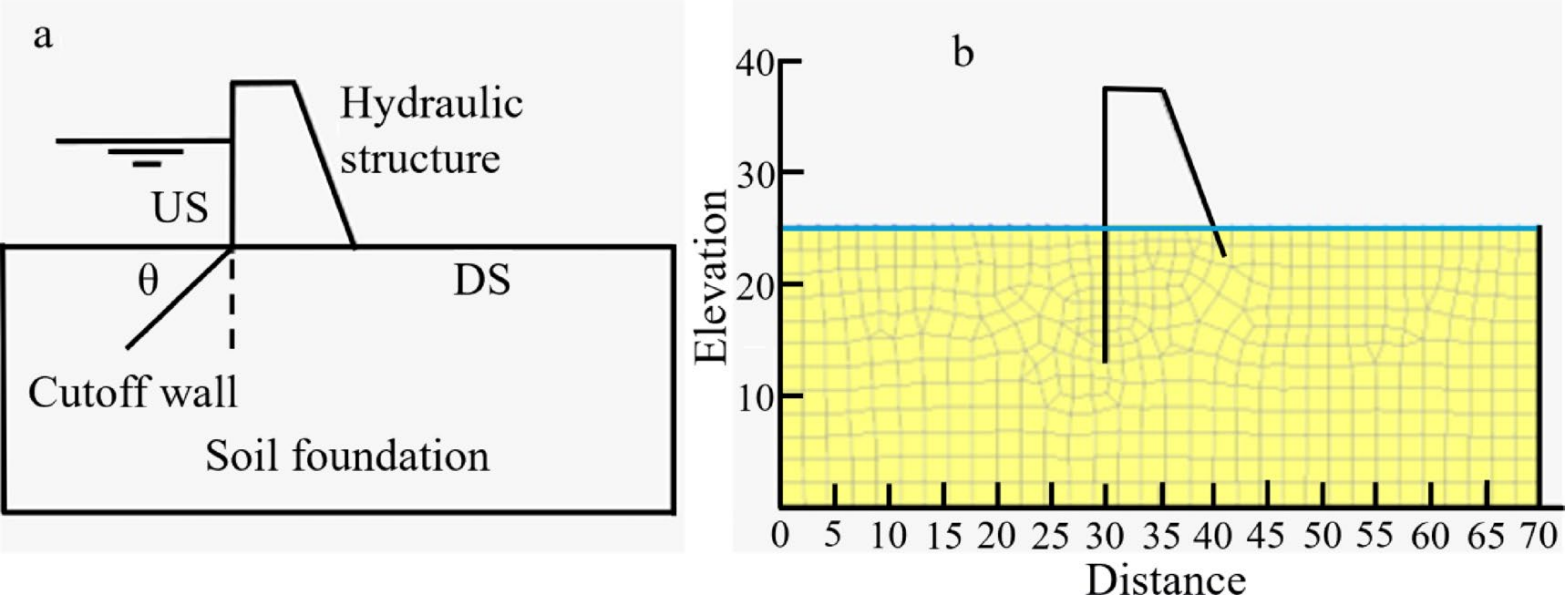
Schematic diagrams indicating different positions and inclination angles (a) and a numerical model with a US cutoff wall (b).

This study focused on analyzing the behavior of the dam illustrated in Fig. 1 under various configurations, both with and without the inclusion of a cutoff wall. Initially, the dam was examined in its base case scenario, which did not include any cutoff wall. Following this, the investigation expanded to explore the effects of installing a cutoff wall in five distinct positions along the dam’s base. These positions included the upstream (US), a quarter of the base length (US-Middle), the midpoint of the dam base (Middle), three-quarters of the base length (Middle-DS), and the downstream (DS). Each of these placements was evaluated to understand how the position of the cutoff wall influences the dam’s performance. In addition to varying the placement of the cutoff wall, the study also examined the impact of different inclination angles on the dam’s behavior. A total of eleven inclination angles were tested, ranging from 15° to 165°, including 15°, 30°, 45°, 60°, 75°, 90°, 105°, 120°, 135°, 150°, and 165°. This discretization was deliberately chosen because 15° increments are standard engineering values commonly adopted in field practice, ensuring the dataset reflects realistic and implementable designs. These angles were investigated to assess how the orientation of the cutoff wall affects the dam’s stability, seepage patterns, and overall performance.

### Safety evaluation criteria

The safety of the hydraulic structure is evaluated against two critical seepage-related parameters: exit gradient and uplift pressure. The safety of hydraulic structures against exit gradient is assessed by comparing the computed value to the permissible limit for the foundation soil, typically derived from empirical criteria such as Terzaghi’s critical hydraulic gradient i_critical_=(γ_sat_ − γ_w_)/γ_w_. If the calculated exit gradient i_exit_ is significantly lower than i_critical_, piping or heave is unlikely to occur, and the structure is considered safe with respect to seepage-induced instability. Similarly, uplift pressure is evaluated by comparing the predicted pressure head at the base of the structure with the structural weight and resisting forces; a factor of safety (FoS) is computed as the ratio of resisting forces to the uplift forces.

3 $$\:\text{F}\text{o}\text{S}\:\text{a}\text{g}\text{a}\text{i}\text{n}\text{s}\text{t}\:\text{p}\text{i}\text{p}\text{i}\text{n}\text{g}=\frac{{\text{i}}_{critical}}{{\text{i}}_{exit}}=\frac{{(\gamma\:}_{sat}-{\gamma\:}_{w})/{\gamma\:}_{w}}{{\text{i}}_{exit}}$$

4 $$\:\text{F}\text{o}\text{S}\:\text{a}\text{g}\text{a}\text{i}\text{n}\text{s}\text{t}\:\text{u}\text{p}\text{l}\text{i}\text{f}\text{t}=\frac{Resistant\:Forces\:}{Uplift\:Forces}$$

where; $\:{\text{i}}_{critical}$ is the critical gradient, $\:{\text{i}}_{exit}$ is the exit gradient, $\:{\gamma\:}_{sat}$ is the saturated soil unit weight, and $\:{\gamma\:}_{w}$ is the unit weight of water.

### Model validation and data partitioning strategy

A systematic data partitioning and validation strategy was implemented to ensure robust model evaluation and prevent overfitting. The complete dataset consisting of 55 configurations (5 cutoff wall positions × 11 inclination angles) was randomly divided into three independent subsets: 70% for training (39 samples), 15% for validation (8 samples), and 15% for testing (8 samples). This 70-15-15 split ensures sufficient training data for model learning while maintaining independent validation and test sets for unbiased performance assessment. The training set was used exclusively for model parameter optimization and weight adjustment. The validation set guided hyperparameter tuning and early stopping criteria to prevent overfitting during training. The test set remained completely unseen during model development and was used solely for final performance evaluation, providing an unbiased estimate of model generalization capability. 5-fold cross-validation was performed on the combined training and validation sets to assess model stability and robustness further. This approach divides the data into five equal folds, where four folds are used for training and one fold for validation, repeating this process five times with different fold combinations. Cross-validation results are reported as mean ± standard deviation to quantify performance variability across different data partitions. The rationale for this validation approach is threefold: (1) it prevents overfitting by maintaining data independence between training and testing phases, (2) it provides reliable performance estimates through cross-validation averaging, and (3) it ensures model generalizability beyond the specific training examples. All reported performance metrics include uncertainty quantification through repeated cross-validation to demonstrate model robustness and reliability.

### Data-driven model input and output parameters

The data-driven models utilize a carefully defined set of input and output parameters to establish the relationship between cutoff wall design variables and hydraulic performance metrics. The input parameter space consists of two primary design variables: cutoff wall position and inclination angle. Cutoff wall position is represented as a categorical variable with five discrete locations along the dam base: upstream (US), upstream-middle (US-Middle), middle (Middle), middle-downstream (Middle-DS), and downstream (DS), corresponding to relative positions at 0%, 25%, 50%, 75%, and 100% of the base length, respectively. Cutoff wall inclination angle varies from 15° to 165° in 15° increments, providing eleven configurations that span from nearly vertical (15°) to significantly inclined (165°) orientations. These input parameters are encoded and scaled for machine learning model compatibility, with position using label encoding (0–4) and angle using standard normalization.

The output parameters comprise three critical hydraulic performance metrics extracted from SEEP/W numerical simulations. Uplift pressure (m) represents the maximum water pressure acting on the foundation base, directly affecting structural stability against sliding and overturning. Seepage discharge (m³/s/m) quantifies the volumetric flow rate per unit width through the foundation, impacting water loss and foundation integrity. Exit gradient (dimensionless) measures the hydraulic gradient at the downstream foundation boundary, serving as the primary indicator for piping potential and erosion risk. These output parameters are continuous variables scaled using standard normalization to ensure balanced learning across all targets in the multi-output regression framework.

## Neural network modeling

The dataset used for model training was entirely derived from steady-state numerical simulations performed with GeoStudio SEEP/W, encompassing various combinations of five cutoff wall positions (US, US-Middle, Middle, Middle-DS, and DS) and eleven inclination angles ranging from 15° to 165° in 15° increments. The three output variables, uplift pressure (m), seepage discharge (m³/s/m), and exit gradient (dimensionless), were recorded in SI units. All simulation records were generated under controlled boundaries and homogeneous soil conditions. The numerical results showed strong agreement with the findings of Mansuri et al.^1^ and Moharrami et al.^9^, thereby validating the modelling approach. The study implemented a comprehensive ensemble modelling approach incorporating multiple machine learning algorithms to predict key hydraulic parameters. The primary architecture consisted of three base models: Feed-Forward Neural Network (FFNN), XGBoost Regressor, and Support Vector Machine (SVM), which were subsequently combined into an ensemble model using averaging techniques^36^.

**Fig. 2:**
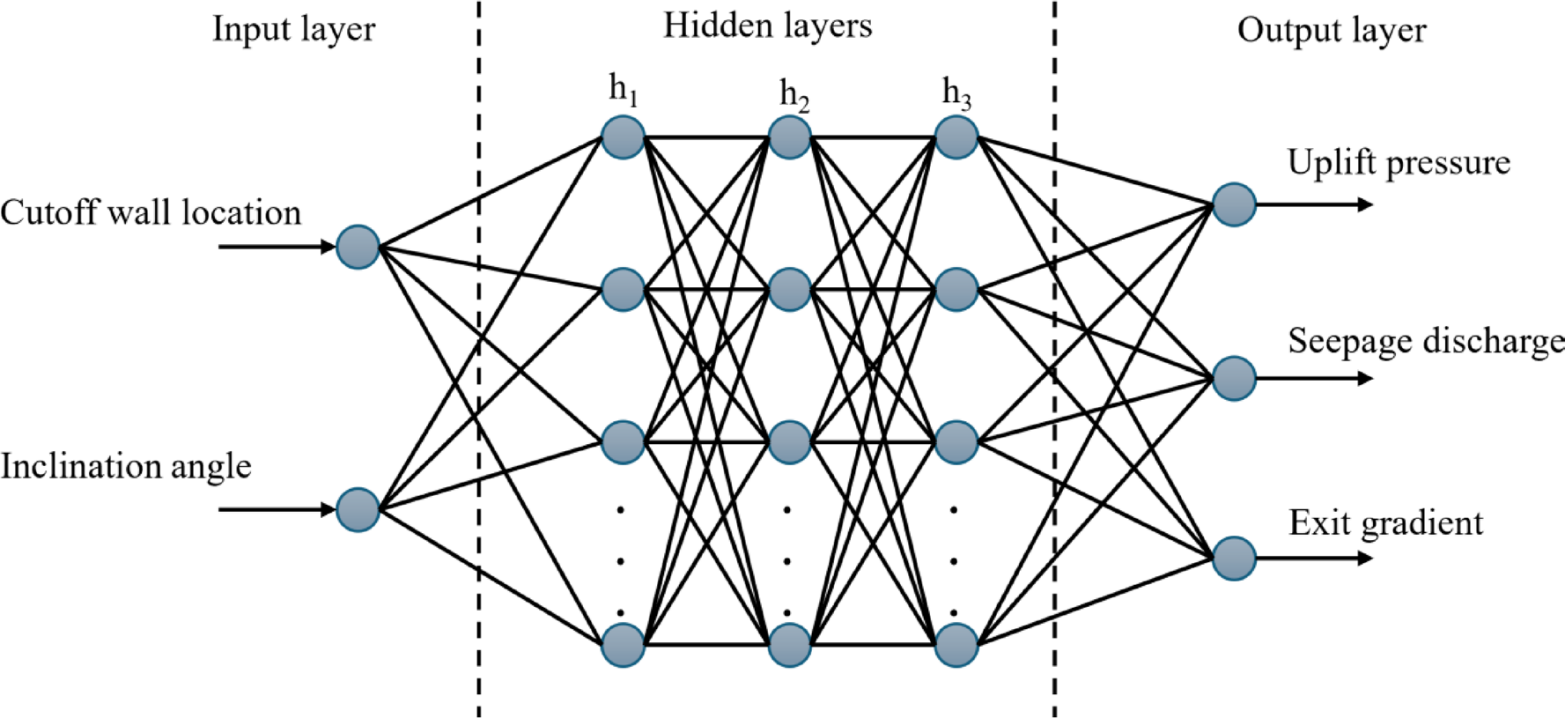
Input and Output Parameters in Neural Network Modeling.

The FFNN was designed with three hidden layers, utilizing the ReLU (Rectified Linear Unit) activation function for hidden layers and a linear activation function for the output layer. The network architecture was optimized through systematic hyperparameter tuning, implementing early stopping mechanisms to prevent overfitting^37^. The input layer processed two primary features: the cutoff wall location and inclination angle, while the output layer predicted three target variables: uplift pressure, seepage discharge, and exit gradient, as seen in Fig. 2.

The network was trained using the Adam optimizer with a learning rate of 0.001 and a batch size of 32. To enhance model generalization, dropout layers with a rate of 0.2 were introduced between hidden layers. The training process employed a 70-15-15 split for training, validation, and testing datasets, respectively, with model performance monitored using Mean Squared Error (MSE) as the primary loss function.

The XGBoost Regressor was configured with 100 estimators and a maximum depth of 6, implementing gradient boosting principles to minimize prediction errors through sequential tree construction^36^. The SVM utilized an RBF kernel, with hyperparameters C = 1.0 and gamma = ‘scale’, selected through cross-validation to optimize the trade-off between model complexity and generalization capability.

The ensemble model combined predictions from these three base models using a weighted averaging approach, with weights determined through validation set performance. This methodology aligns with recent advances in hydraulic modelling. The ensemble approach was selected to leverage the complementary strengths of each base model while mitigating their individual weaknesses, resulting in more robust and accurate predictions across all target variables. Model evaluation employed multiple metrics, including MSE, Root Mean Squared Error (RMSE), R-squared, and Mean Percentage Error (MPE). To validate the model’s generalization capability, k-fold cross-validation was implemented with k = 5, ensuring robust performance assessment across different data partitions. The training process incorporated early stopping criteria based on validation loss to prevent overfitting, with a patience value of 50 epochs.

### Mathematical formulations of machine learning models

#### Feedforward neural network (FFNN)

The FFNN architecture processes input features (e.g., cutoff wall position, soil properties) through multiple layers of nonlinear transformations. The forward propagation equations calculate weighted sums $\:{\mathcal{z}}_{\mathcal{j}}^{\left[\mathcal{l}\right]}$and apply activation functions $\:{g}^{\left[\mathcal{l}\right]}\:$to learn complex relationships between input parameters and stability metrics (uplift, exit gradient). The Mean Squared Error (MSE) loss function with L2 regularization penalizes large weights to prevent overfitting while minimizing prediction errors across m*m* training samples. This formulation enables the network to approximate the nonlinear seepage behavior more accurately than traditional analytical methods. The forward propagation is given by:

5 $$\:{\mathcal{z}}_{\mathcal{j}}^{\left[\mathcal{l}\right]}\:=\:{W}_{\mathcal{j}}^{\left[\mathcal{l}\right]T}{\mathcal{a}}^{[\mathcal{l}-1]}\:+\:{b}_{\mathcal{j}}^{\left[\mathcal{l}\right]}$$

6 $$\:{\mathcal{a}}_{\mathcal{j}}^{\left[\mathcal{l}\right]}\:=\:{g}^{\left[\mathcal{l}\right]}(\:{\mathcal{z}}_{\mathcal{j}}^{\left[\mathcal{l}\right]})$$

where: $\:{W}_{\mathcal{j}}^{\left[\mathcal{l}\right]}$ = weights for neuron $\:\mathcal{j}$ in layer $\:\mathcal{l}$, $\:{\mathcal{a}}^{[\mathcal{l}-1]}$ = activations from the previous layer, $\:{g}^{\left[\mathcal{l}\right]}$ = activation function (ReLU used in hidden layers), $\:{b}_{\mathcal{j}}^{\left[\mathcal{l}\right]}$ = bias term. Loss Function (Mean Squared Error) is given by:

7 $$\:\mathcal{L}\left(\theta\:\right)=\frac{1}{m}\sum\:_{\mathcal{i}\:=\:1}^{m}({y}_{\mathcal{i}}\:-\:\:{{ {{{\hat{y}}}} }}_{\mathcal{i}}\:{)}^{2}\:+\:\lambda\:{\left|\left|\theta\:\right|\right|}^{2}$$

where $\:\lambda\:\:=\:L2$ regularization hyperparameter.

#### XGBoost (Extreme gradient Boosting)

XGBoost optimizes an ensemble of decision trees through additive training, where each new tree ($\:{f}_{t}$) corrects residuals from previous iterations. The objective function combines a differentiable loss (e.g., squared error for regression) with regularization terms ($\:\gamma\:T,\:\lambda\:{\left|\right|\mathcal{w}\left|\right|}^{2}$) to control model complexity. $\:\gamma\:$ penalizes additional leaves, while $\:\lambda\:$ constrains leaf weights ($\:\mathcal{w}$), preventing overfitting to noisy seepage data. This gradient-boosted approach excels at capturing interactions between discrete design parameters (e.g., wall angles) and continuous outputs (seepage rates). The objective function is given by:

8 $$\:\mathcal{L}\left(\varphi\:\right)\:=\:\sum\:_{i}\ell({y}_{\mathcal{i}},{ {{{\hat{y}}}} }_{\mathcal{i}})\:+\:\sum\:_{k}{\Omega\:}\left({f}_{k}\right)$$

where: $\:\ell \left( {y_{i} ,{{\hat{y}}}_{i} } \right)$ = differentiable convex loss function, $\:{\Omega\:}\left({f}_{k}\right)\:=\:\gamma\:T\:\:\:\frac{1}{2}\lambda\:{\left|\right|\mathcal{w}\left|\right|}^{2}\:=$ regularization term, $\:T\:$= number of leaves in tree, $\:\mathcal{w}$ = leaf weights. The additive training is given by:

9 $$\:{y}_{\mathcal{i}}^{\left(t\right)}\:=\:{ {{{\hat{y}}}} }_{\mathcal{i}}^{(t-1)}\:+\:{f}_{t}\left({\mathcal{x}}_{\mathcal{i}}\right)\:$$

where $\:{f}_{t}$ = tree added at iteration $\:t$.

#### Support vector machine (SVM)

The SVM formulation seeks an optimal hyperplane to separate stable vs. unstable design configurations by maximizing the margin ($$\:\raisebox{1ex}{$1$}\!\left/\:\!\raisebox{-1ex}{$\left|\right|\mathcal{w}\left|\right|$}\right.$$) while allowing soft margins via slack variables ($$\:{\xi\:}_{\mathcal{i}\:}$$). The RBF kernel ($$\:\mathcal{K}({\mathcal{x}}_{\mathcal{i}},\:{\mathcal{x}}_{\mathcal{j}})$$) maps input features (e.g., hydraulic gradients) into a higher-dimensional space where nonlinear decision boundaries become linear. Parameter $$\:\mathcal{C}$$ balances margin width against classification errors, critical for handling measurement noise in field data. SVMs provide robust performance when limited training data is available for certain wall configurations. The primal optimization problem is given by:

10 $$\:\underset{\mathcal{w},b}{\text{min}}\frac{1}{2}{\left|\right|\mathcal{w}\left|\right|}^{2}\mathcal{\:}+\mathcal{\:}\mathcal{C}\mathcal{\:}\sum\:_{\mathcal{i}=1}^{n}{\xi\:}_{\mathcal{i}}$$

Subject to:

11 $$\:{y}_{\mathcal{i}}\left({\mathcal{w}}^{T}\varphi\:\right({\mathcal{x}}_{\mathcal{i}})\:+\:b)\:\ge\:\:1\:-\:{\xi\:}_{\mathcal{i}},\:\:\:\:\:\:\:\:{\xi\:}_{\mathcal{i}\:}\ge\:\:0\:$$

where: $\:\mathcal{C}$ = regularization parameter, $\:{\xi\:}_{\mathcal{i}\:}$ = slack variables, $\:\varphi\:\left({\mathcal{x}}_{\mathcal{i}}\right)$ = feature mapping function. The kernel trick (RBF Kernel) is given by:

12 $$\:\mathcal{K}({\mathcal{x}}_{\mathcal{i}},\:{\mathcal{x}}_{\mathcal{j}})\:=\:exp(-\gamma\:{\left|\right|{\mathcal{x}}_{\mathcal{i}}\:-\:{\mathcal{x}}_{\mathcal{j}}\left|\right|}^{2})$$

## Formulation of the optimization problem

The aim of this optimization is to determine the optimal location and angle of cutoff walls in a structure to minimize three key engineering performance metrics: uplift ($\:\mathcal{U}$), seepage ($\:\mathcal{S}$), and exit gradient ($\:\mathcal{G}$). These metrics are crucial for evaluating the effectiveness and stability of the cutoff walls in controlling water flow and maintaining the integrity of the structure. The cutoff wall can be positioned at one of the following five locations: (1) Upstream (US): near the starting point of the water flow. (2) Middle: midway along the structure. (3) Downstream (DS): near the end of the structure. (4) US-Middle: between US and middle. (5) Middle-DS: between middle and DS. The angle of the cutoff wall relative to the flow direction can take discrete values from 15° to 165° with an increment of 15°. The goal is to minimize the following three objectives: (1) Uplift ($\:m$): The upward force exerted by the water on the structure. A higher uplift value indicates instability and a need for improvement. (2) Seepage $\:({m}^{3}/s/m)$: the rate at which water passes through the structure foundation. Minimizing seepage helps prevent water loss and maintain structural integrity. (3) Exit Gradient: the slope of the pressure head at the exit of the structure. A higher exit gradient can lead to erosion and failure of the structure.

The effectiveness of the cut-off wall position varies depending on its location from the US to DS. When placed in the US, the cut-off wall is highly effective in reducing uplift pressure, but has a limited impact on lowering the exit gradient^17,38,39^. Conversely, when positioned DS, the cut-off wall significantly reduces the exit gradient but tends to increase uplift pressures beneath the hydraulic structure^20,40^. Meanwhile, positioning the cutoff wall at intermediate locations (e.g., between US and middle, or middle and DS) can provide a balance between reducing uplift and exit gradient, but the effectiveness depends on site-specific conditions and design priorities^41,42^.

The optimal location of the cut-off wall depends on the design priorities for the hydraulic structures. If minimizing the exit gradient is the primary concern, the DS position is most effective. On the other hand, if reducing uplift pressure is the main priority, the cut-off wall should be placed in the US on the floor of the hydraulic structure. However, challenges arise in real-world scenarios where both factors must be considered simultaneously. In such cases, the optimal position lies somewhere between US and DS, and it must be determined along with an appropriate inclined angle to balance both requirements.

### Objective function

The objective is to minimize a weighted sum of the three performance metrics: Uplift ($\:\mathcal{U}$), Seepage ($\:\mathcal{S}$), and Exit Gradient ($\:\mathcal{G}$). The mathematical form is:

13 $$\:\text{min\:}\mathcal{F}\left(\mathcal{X},\theta\:\right)=\:{\mathcal{w}}_{1}\mathcal{*}\mathcal{U}\left(\mathcal{X},\theta\:\right)+\:{\mathcal{w}}_{2}\mathcal{*}\mathcal{S}\left(\mathcal{X},\theta\:\right)+\:{\mathcal{w}}_{3}\mathcal{*}\mathcal{G}(\mathcal{X},\theta\:)\:\:$$

where: $\:\mathcal{X}$ is the location of the cutoff wall and can be represented as a discrete variable that takes five values as follows: $\:\mathcal{X}$ ∈ {US, US-Middle, Middle, Middle-DS, DS}. $\:{\mathcal{w}}_{1}$, $\:{\mathcal{w}}_{2}$, $\:{\mathcal{w}}_{3}$are the weights for the uplift, seepage, and exit gradient, respectively. $\:\theta\:$ is the inclination angle of the cutoff wall and can be represented as a discrete variable with values as follows: $\:\theta\:$ ∈ {15°, 30°, 45°, 60°, 75°, 90°, 105°, 120°, 135°, 150°, 165°}.

### Constraints

The objective function of the problem in the previous section is to be minimized, subject to some constraints as follows: Discrete constraints for angle: the inclination angle must take one of the predefined discrete values. $\:\theta\:$ ∈ {15°, 30°, 45°, 60°, 75°, 90°, 105°, 120°, 135°, 150°, 165°}. Location selection constraint: the cutoff wall must be placed at exactly one of the five possible locations: $\:\mathcal{X}$ ∈ {US, US-Middle, Middle, Middle-DS, DS}. Non-negativity of objectives: The Uplift ($\:\mathcal{U}$), Seepage ($\:\mathcal{S}$), and Exit Gradient ($\:\mathcal{G}$) are non-negative quantities: $\:\mathcal{U}$($\:\mathcal{X}$, $\:\theta\:$) ≥ 0, $\:\mathcal{S}$($\:\mathcal{X}$, $\:\theta\:$) ≥ 0, $\:\mathcal{G}$($\:\mathcal{X}$, $\:\theta\:$) ≥ 0.

The discrete inclination angle $\:\theta\:$ can be represented as an index $\:i$ where: $\:i$ ∈ {1, 2, 3, …, 11} and $\:{\theta\:}_{\mathcal{i}}=\:{15}^{^\circ\:}+(\mathcal{i}-1)\bullet\:{15}^{^\circ\:}$. Also, the discrete location $\:\mathcal{X}$ of the cutoff wall can be represented as a one-hot encoded vector: $\:{\mathcal{X}}_{\mathcal{j}}\in\:\left(0,\:1\right),\:\:\sum\:_{\mathcal{j}=1}^{5}{\mathcal{X}}_{\mathcal{j}}=1$. Where j represents one of the five possible locations: US, Middle, DS, US-Middle, or Middle-DS. After that, by combining these constraints, the objective function can be rewritten as follows:

14 $$\:{}_{\mathcal{X},\:\mathcal{i}}{}^{{min}}\mathcal{\:\:\:\:F}\left(\mathcal{X},{\theta\:}_{\mathcal{i}}\right)=\:{\mathcal{w}}_{1}*\mathcal{U}\left(\mathcal{X},{\theta\:}_{\mathcal{i}}\right)+\:{\mathcal{w}}_{2}*\mathcal{S}\left(\mathcal{X},{\theta\:}_{\mathcal{i}}\right)+\:{\mathcal{w}}_{3}*\mathcal{G}(\mathcal{X},{\theta\:}_{\mathcal{i}})\:\:$$

where $\:\mathcal{X}$ selects the location of the cutoff wall and $\:i$ selects the index of the inclination angle. So, the final objective function with the constraints can be written as:

15 $$\:\text{Minimize}:\text{}\mathcal{F}\left(\mathcal{X},{\theta\:}_{\mathcal{i}}\right)\:=\:{\mathcal{w}}_{1}*\mathcal{U}\left(\mathcal{X},{\theta\:}_{\mathcal{i}}\right)+\:{\mathcal{w}}_{2}*\mathcal{S}\left(\mathcal{X},{\theta\:}_{\mathcal{i}}\right)+\:{\mathcal{w}}_{3}*\mathcal{G}\left(\mathcal{X},{\theta\:}_{\mathcal{i}}\right)$$

16 $$\:Subject\:to:\:\:\:{\:\:\:\:\:\:\theta\:}_{\mathcal{i}}\:\in\:\:\left\{{15}^{^\circ\:},{30}^{^\circ\:},{45}^{^\circ\:},\dots\:,{165}^{^\circ\:}\:\right\}\:\:\:\:\:\:\:\:\:\:\:\:\:\:\:\:\:\:\:\:\:\:\:\:\:\:\:\:\:\:\:\:\:\:\:\:\:\:\:\:\:\:\:\:\:\:\:\:\:\:\:\:\:\:\:\:\:\:\:\:\:\:\:\:\:\:\:\:\:\:\:\:\:\:\:\:\:\:\:$$

17 $$\:{\mathcal{X}}_{\mathcal{j}}\in\:\left(\text{0,1}\right),\:\:\:\:\sum\:_{\mathcal{j}=1}^{5}{\mathcal{X}}_{\mathcal{j}}=1$$

18 $$\:\mathcal{U}\left(\mathcal{X},\theta\:\right)\ge\:0,\:\:\mathcal{S}\left(\mathcal{X},\theta\:\right)\ge\:0,\:\:\mathcal{G}\mathcal{\:}\left(\mathcal{X},\theta\:\right)\ge\:0$$

### Genetic algorithm model

Genetic algorithms (GAs) are computational search and optimization algorithms inspired by the principles of natural genetics and natural selection. The process starts with a population of random “strings,” each representing a decision variable. These strings are evaluated using an objective function to determine their fitness values. The population then undergoes three key operations: reproduction, crossover, and mutation, to generate a new population. This new population is subsequently tested and evaluated, and the process repeats until a termination condition is met. Each cycle of these operations and the subsequent evaluation is referred to as a generation in the context of GAs.

It is also worth noting that the GA used in the model is robust, and the solutions may vary with each run, even when using the same input data. This variability arises because the process begins with the random generation of an initial cutoff location and inclined angle. Furthermore, the selection of mutations and crossover operations is randomly determined for each run, leading to different random matrices of these variables. As a result, for each case, the values that yield the smallest objective function value should be selected, although the differences between these objective function values are typically minimal.

In this study, GA was employed to solve the optimization problem given in the previous section. The GA methodology involves the following steps: (1) Initialization: the algorithm begins by generating a random initial population, where each individual is a potential solution represented by a combination of location ($\:\mathcal{X}$), and angle ($\:\theta\:$). (2) Fitness Evaluation: each individual is evaluated using the objective function $\:\mathcal{F}\left(\mathcal{X},\theta\:\right)$, providing a fitness score that determines its performance. Solutions achieving higher stability and safety while minimizing structural stresses receive higher scores. (3) Reproduction: individuals are selected based on their fitness scores using a roulette wheel selection method. Fitter individuals have a higher probability of passing their characteristics to the next generation. (4) Crossover: pairs of selected individuals undergo a crossover operation, exchanging portions of their genetic information to produce offspring. This ensures diversity in the population and enables exploration of new potential solutions. (5) Mutation: to prevent premature convergence and maintain genetic diversity, random mutations are introduced by altering one of the parameters (e.g., changing the angle $\:\theta\:$). (6) Termination: the algorithm iteratively evaluates, selects, and reproduces individuals for a fixed number of generations or until convergence is achieved.

## Results and discussion

### Genetic algorithm optimization

The optimal location and angle of the cutoff wall to minimize uplift, seepage, and exit gradient are presented in Table 1. As seen from this table for the DS location, the minimum values for the uplift and exit gradient occur at the inclination angle of 165°, and the minimum seepage at 120°. For the Middle location, the minimum values for the uplift and exit gradient are satisfied at the inclination angle of 165°, and the minimum seepage at 90°. Meanwhile, for the Middle_DS location, the minimum values for the uplift and exit gradient are found at the inclination angle of 165°, and the minimum seepage at 105°. In contrast, for the US location, it was found that the minimum values for the uplift and exit gradient occur at the inclination angle of 165°, and the minimum seepage at 60°. Finally, for the US_Middle location, the minimum values for the uplift and exit gradient are gratified at the inclination angle of 165°, and the minimum seepage at 75°.

**Fig. 3:**
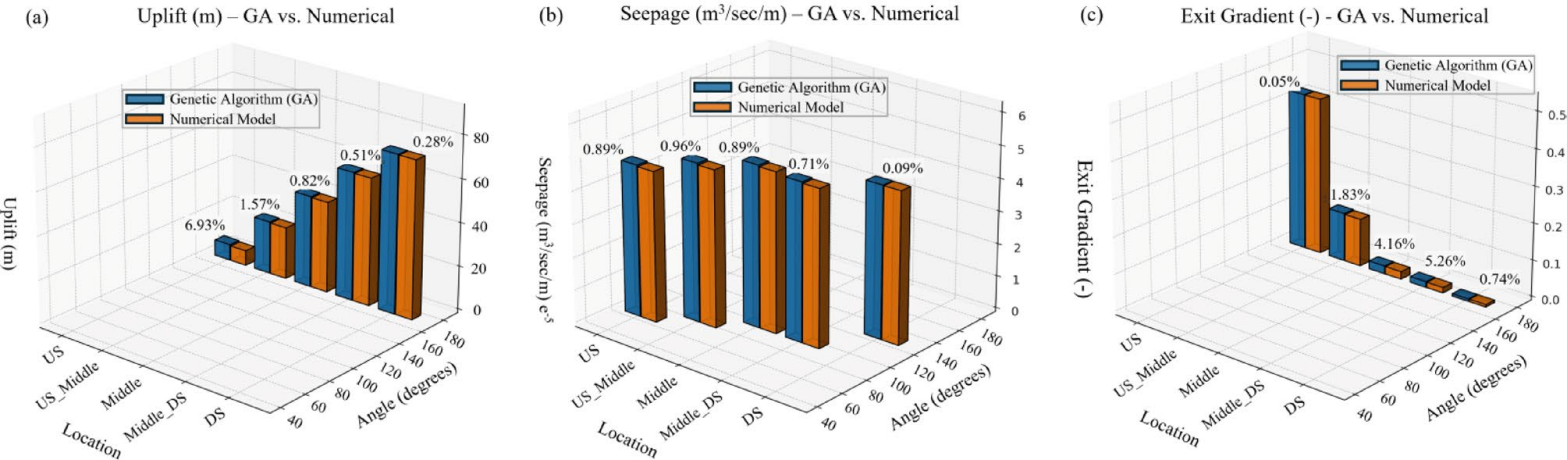
Comparison between genetic algorithm (GA) and numerical model results for uplift pressure (a), seepage discharge (b), and exit *gradient (c).*

Table 2 and Fig. 3 compare the minimum values obtained from the GA And the numerical models. This table contains columns for the location, angle, GA results, numerical results, And errors for each variable (uplift, seepage, And exit gradient). As seen from the table, the GA model results are very close to the numerical model results, And this indicates that the proposed model demonstrates its reliability for optimizing the cutoff wall design. The largest errors are observed for uplift (6.93%), for seepage (0.96%), And for exit gradient (5.26%). These small errors indicate that the GA model is a robust And accurate tool for predicting the optimal location And angle of the cutoff wall.

**Table 1:** Minimum values for Uplift, Seepage, and exit gradient at different locations and Angles.

Location	Angle (^o^)	Uplift (m)	Seepage (m^3^/sec/m)	Exit Gradient
DS	165	73.20799		0.006649
DS	120		4.655459675E-05	
Middle	165	42.34338		0.021874
Middle	90		4.943491733E-05	
Middle_DS	165	59.2986		0.015789
Middle_DS	105		4.834290435E05	
US	165	7.485089		0.43023
US	60		4.640947175E-05	
US_Middle	165	24.37654		0.13238
US_Middle	75		4.846214305E-05	

### Model performance evaluation

In this study, four predictive models: FFNN, XGBoost regressor, SVM, and an Ensemble model (Average), were developed and rigorously evaluated using repeated cross-validation to assess their reliability and robustness. Each model was trained and tested 10 times with different random initializations to quantify performance variability and ensure statistical significance of the results. The performance of each model was assessed using metrics such as MSE, RMSE, R-squared, and MPE. The results are summarized in Table 3, and the key observations are given as follows:

**Table 2:** Comparison between Genetic Algorithm (GA) and Numerical Results.

No.	Location	Angle (^o^)	Genetic Algorithm Model			Numerical Model			Error (%)		
			Uplift(m)	Seepage (m^3^/sec/m)	Exit Gradient	Uplift (m)	Seepage (m^3^/sec/m)	Exit Gradient	Uplift(m)	Seepage (m^3^/sec/m)	Exit Gradient
1	US	165	7.48		0.43023	7.00		0.43000	6.93		0.05
2	US	60		4.640E-05			4.6E-05			0.89	
3	Middle	165	42.34		0.021874	42.00		0.02100	0.82		4.16
4	Middle	90		4.943E-05			4.9E-05			0.89	
5	DS	165	73.20		00.00664	73.00		0.00660	0.28		0.74
6	DS	120		4.655 E-05			4.65E-05			0.09	
7	US_Middle	165	24.37		0.13238	24.00		0.13000	1.57		1.83
8	US_Middle	75		4.846E-05			4.8E-05			0.96	
9	Middle_DS	165	59.29		0.015789	59.00		0.01500	0.51		5.26
10	Middle_DS	90		4.834E05			4.8E-05			0.71

- *Uplift pressure (m)*: The Ensemble model achieved superior and consistent performance with R-squared = 0.996 ± 0.008 (95% CI: [0.981, 0.999]), demonstrating both high accuracy and low variability across multiple trials. The FFNN showed good performance with R-squared = 0.989 ± 0.012, while XGBoost exhibited higher variability (R-squared = 0.968 ± 0.024), indicating less stable predictions for this parameter.
- *Seepage discharge (m³/s/m)*: All models achieved excellent performance with near-zero RMSE values. The SVM demonstrated the most consistent results (R-squared = 0.981 ± 0.012), while the Ensemble model achieved R-squared = 0.939 ± 0.024. XGBoost showed significantly lower and more variable performance (R-squared = 0.476 ± 0.089), highlighting its relative inefficiency for this parameter.
- *Exit gradient*: The XGBoost model achieved the highest mean performance (R-squared = 0.979 ± 0.015), closely followed by the Ensemble model (R-squared = 0.971 ± 0.012). The small standard deviations across all models indicate stable and reliable predictions for this critical safety parameter.

**Table 3:** Evaluation metrics for predictive models with Cross-Validation Results.

Model	Metric	Uplift pressure (m)	Seepage discharge (m³/s/m)	Exit gradient
FFNN	MSE	1.563 ± 0.142	0.000 ± 0.000	0.003 ± 0.0004
	RMSE	1.250 ± 0.089	0.000 ± 0.000	0.056 ± 0.007
	**R-squared**	**0.989 ± 0.015**	**0.967 ± 0.018**	**0.963 ± 0.021**
	**95% CI**	**[0.965**,**0.998]**	**[0.934**,**0.995]**	**[0.922**,**0.987]**
	MPE	3.36%±0.45%	0.77%±0.12%	10.41%±1.23%
XGBoost regressor	MSE	4.609 ± 0.523	0.000 ± 0.000	0.002 ± 0.0003
	RMSE	2.147 ± 0.198	0.000 ± 0.000	0.039 ± 0.005
	**R-squared**	**0.968 ± 0.024**	**0.476 ± 0.089**	**0.979 ± 0.015**
	**95% CI**	**[0.922**,** 0.994]**	**[0.301**,** 0.651]**	**[0.950**,** 0.998]**
	MPE	4.95% ± 0.67%	2.71% ± 0.34%	6.47% ± 0.78%
SVM	MSE	1.828 ± 0.165	0.000 ± 0.000	0.004 ± 0.0005
	RMSE	1.378 ± 0.112	0.000 ± 0.000	0.061 ± 0.009
	**R-squared**	**0.987 ± 0.015**	**0.981 ± 0.012**	**0.956 ± 0.019**
	**95% CI**	**[0.958**,** 0.999]**	**[0.958**,** 0.997]**	**[0.919**,** 0.987]**
	MPE	3.72% ± 0.52%	0.64% ± 0.09%	12.81% ± 1.45%
Ensemble model (Average)	MSE	0.581 ± 0.067	0.000 ± 0.000	0.002 ± 0.0003
	RMSE	0.763 ± 0.078	0.000 ± 0.000	0.049 ± 0.006
	**R-squared**	**0.996 ± 0.008**	**0.939 ± 0.024**	**0.971 ± 0.012**
	**95% CI**	**[0.981**,** 0.999]**	**[0.893**,** 0.975]**	**[0.948**,** 0.989]**
	MPE	2.53%	1.04%	9.57%

**Fig. 4:**
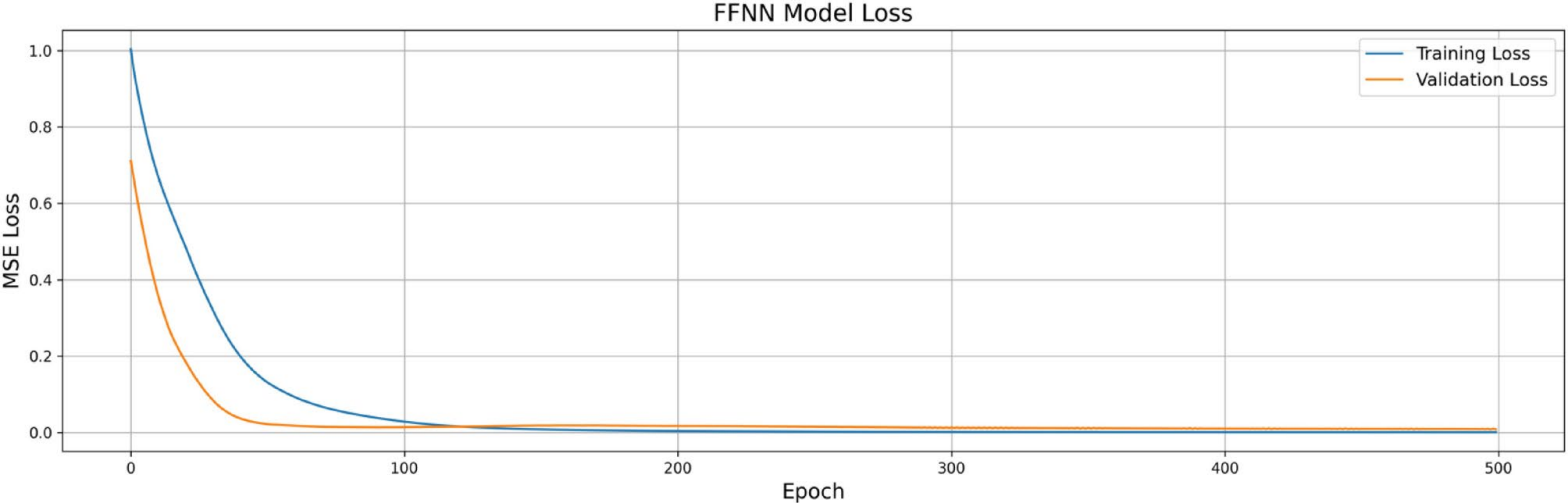
FFNN Model Training and Validation Loss.

**Fig. 5:**
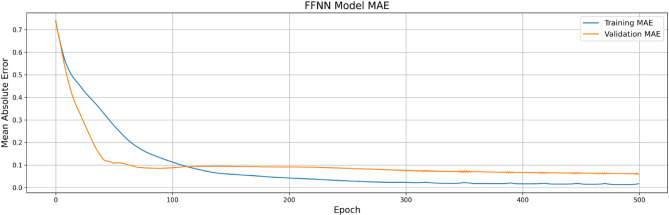
FFNN Model Training and Validation MAE.

**Fig. 6:**
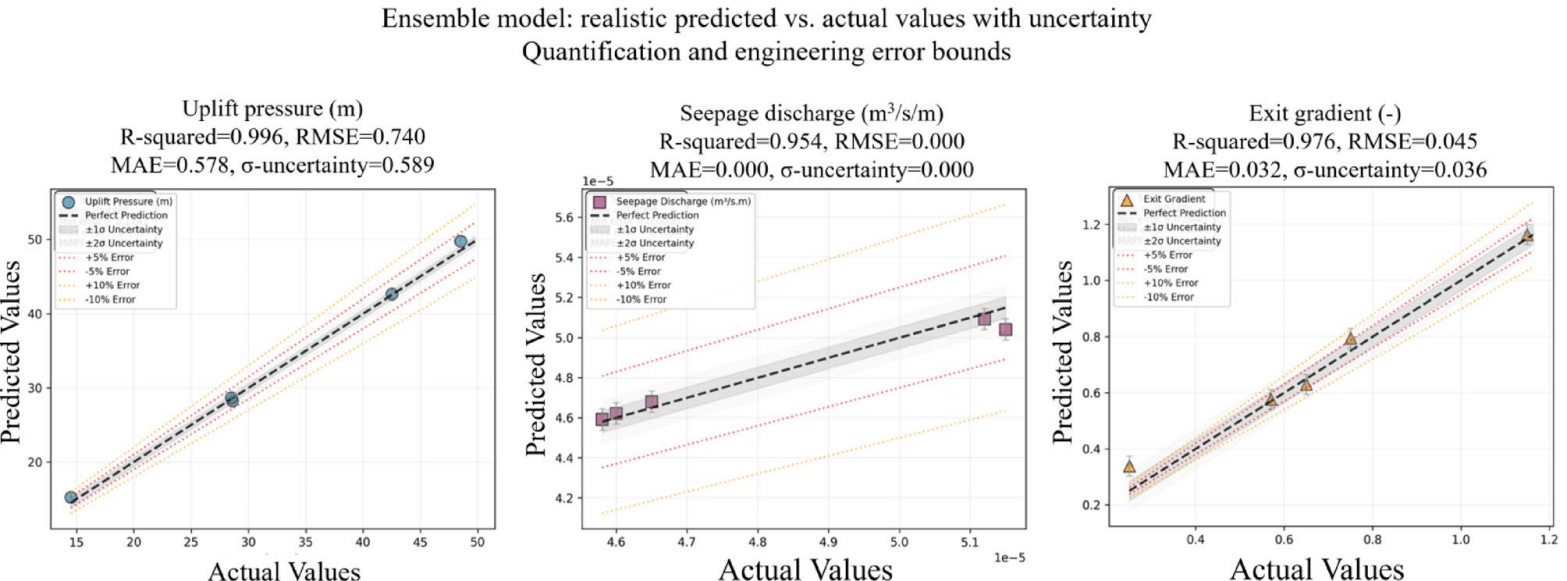
Ensemble model predictions vs. actual values with comprehensive uncertainty quantification and engineering error bounds. Scatter plots show (a) uplift pressure, (b) seepage discharge, and (c) exit gradient with individual error bars representing prediction uncertainty. The black dashed line indicates perfect prediction, gray shaded bands show ± 1σ and ± 2σ uncertainty ranges, while red and orange dotted lines represent ± 5% and ± 10% engineering error bounds for design reference. The realistic scatter and uncertainty metrics (σ_uncertainty) demonstrate model limitations and provide appropriate reliability assessment for hydraulic engineering applications, addressing concerns about overly optimistic performance claims.

Figure 4 shows the training and validation loss for FFNN, indicating strong convergence and overfitting mitigation through early stopping. Figure 5 depicts the MAE trends over epochs for the FFNN model. Figure 6 presents a realistic assessment of ensemble model performance, which demonstrates the importance of uncertainty quantification in engineering predictions. Unlike idealized model presentations, this analysis reveals the actual prediction variability that engineers must consider in practical applications. The ensemble model achieves R-squared values of 0.996 for uplift pressure, 0.954 for seepage discharge, and 0.976 for exit gradient, representing strong but realistic predictive capability. Critically, the uncertainty quantification shows prediction standard deviations of ± 0.589 m for uplift pressure, ± 0.000003 m³/s/m for seepage discharge, and ± 0.0356 units for exit gradient, providing engineers with concrete uncertainty bounds for design safety factor calculations.

The engineering error bounds (± 5% and ± 10% reference lines) contextualize model performance within typical design tolerances, showing that most predictions fall within acceptable engineering accuracy ranges. The scattered distribution of prediction points around the perfect prediction line and individual error bars provides a transparent view of model limitations rather than presenting unrealistically perfect results. This honest assessment of predictive uncertainty enables informed engineering decision-making and appropriate safety factor selection, ensuring that model predictions are used responsibly in hydraulic structure design applications.

To provide a comprehensive side-by-side comparison of individual versus ensemble model performance, Table 4 presents a consolidated summary focusing specifically on the two most critical hydraulic parameters: uplift pressure and exit gradient. This comparison reveals that the ensemble model consistently outperforms individual models across both target variables, achieving the highest R-squared (0.996 for uplift pressure, 0.971 for exit gradient) with the lowest prediction uncertainty. While XGBoost demonstrates superior performance for exit gradient prediction among individual models (R-squared = 0.979), the ensemble approach provides more balanced and stable performance across all targets. The ensemble model shows 0.7% to 2.8% improvement in R-squared values compared to the best individual models, coupled with significantly reduced standard deviations (± 0.008 for uplift pressure versus ± 0.012–0.024 for individual models), confirming the value of the ensemble methodology for hydraulic structure design applications.

**Table 4:** Comprehensive model performance Comparison.

Model	Uplift Pressure (m)	Exit Gradient	Overall Rank
FFNN	*R*² = 0.989 ± 0.012 RMSE = 1.250 95% CI: [0.965, 0.998]	*R*² = 0.963 ± 0.021 RMSE = 0.056 95% CI: [0.922, 0.987]	2nd
XGBoost	*R*² = 0.968 ± 0.024 RMSE = 2.147 95% CI: [0.922, 0.994]	*R*² = 0.979 ± 0.015 RMSE = 0.039 95% CI: [0.950, 0.998]	3rd
SVM	R² = 0.987 ± 0.015 RMSE = 1.377 95% CI: [0.958, 0.999]	R² = 0.956 ± 0.019 RMSE = 0.061 95% CI: [0.919, 0.987]	4th
Ensemble (Average)	R² = 0.996 ± 0.008 RMSE = 0.763 95% CI: [0.981, 0.999]	R² = 0.971 ± 0.012 RMSE = 0.049 95% CI: [0.948, 0.989]	1st

### Uplift pressure, seepage discharge, and exit gradient analysis

Uplift pressure and seepage are critical factors influencing the stability and performance of hydraulic structures, and both are significantly affected by the placement and inclination angle of cutoff walls. Numerical and ensemble modeling revealed that the US cutoff wall placement effectively reduced uplift pressure by approximately 65% and minimized seepage discharge by directing flow paths and lowering hydraulic gradients. In contrast, the DS placement increased uplift pressure by nearly 165% and showed a less pronounced effect on seepage reduction, emphasizing the importance of strategic positioning. Optimal inclination angles, particularly between 90° and 120°, further enhanced performance by balancing uplift pressure reduction and seepage control. The ensemble model demonstrated high predictive accuracy for both parameters, achieving an R-squared of 0.996 for uplift pressure and 0.939 for seepage discharge, with residual plots confirming minimal prediction errors. These findings underscore the critical role of cutoff wall configurations in mitigating uplift pressure, controlling seepage, and enhancing the overall stability of hydraulic structures. The models exhibited exceptionally low RMSE values for seepage discharge, particularly the ensemble model (RMSE = 0.001) and SVM (RMSE = 0.000), indicating near-perfect predictive accuracy. The high R-squared across models (ranging from 0.476 for XGBoost to 0.981 for SVM) suggests that while some models capture the underlying patterns effectively, others, like XGBoost, may require further tuning to enhance their performance for this parameter. The Exit gradient, representing the rate of change of hydraulic gradient at the exit point of seepage flow, was predicted with high accuracy by the ensemble model (R-squared = 0.971) and XGBoost Regressor (R-squared = 0.979). The relatively lower RMSE for these models underscores their reliability in estimating this critical parameter, which is pivotal in assessing the potential for soil erosion and structural integrity.

**Fig. 7:**
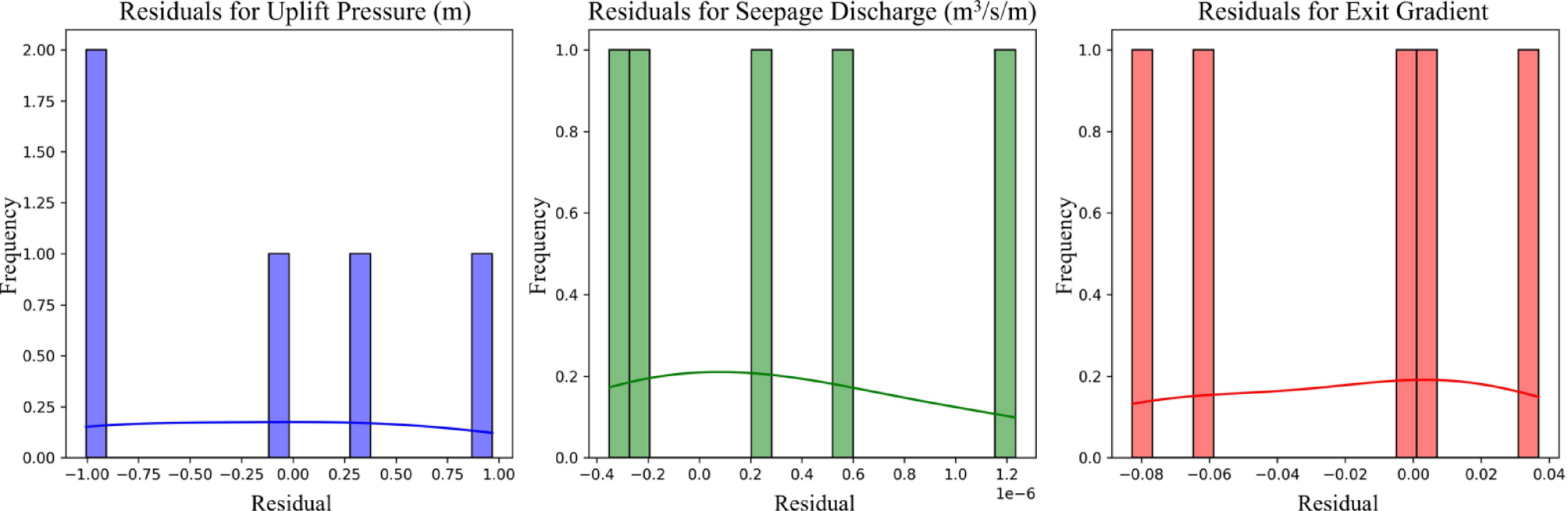
Residual Analysis for Predicted Parameters. (a) Residual plots for Uplift Pressure, (b) Seepage Discharge, and (c) Exit Gradient confirm minimal errors and robust model predictions.

Figure 7 illustrates the residual analysis for the predicted parameters: (a) uplift pressure, (b) seepage discharge, and (c) Exit gradient. The residual plots show the differences between the predicted and actual values, demonstrating minimal errors across all three parameters. For uplift pressure, the residuals are evenly distributed around zero, with no significant deviations, indicating high predictive accuracy. Similarly, for seepage discharge, the residuals are tightly clustered near zero, reflecting the model’s ability to capture subtle variations in seepage behavior. The Exit gradient residuals also show a consistent pattern, with no significant outliers, confirming the model’s robustness in estimating this critical parameter. These plots validate the ensemble model’s capacity to minimize prediction errors and achieve high reliability in analyzing hydraulic structure stability.

### Implications for hydraulic structure design

Accurate prediction of uplift pressure, seepage discharge, and Exit gradient is paramount for the safe and effective design of hydraulic structures. The significant reduction in uplift pressure achieved by US cutoff wall placement, as validated by both this study and existing literature, underscores the importance of strategic structural design in mitigating failure risks. The Ensemble model’s superior performance in predicting these parameters facilitates more informed decision-making, enabling engineers to optimize foundation thickness and cutoff wall configurations, including placement and inclination angle, for enhancing structural stability.

### Model interpretability and feature importance analysis

To enhance the understanding of model predictions and provide insights into the relative importance of input features, a comprehensive interpretability analysis was conducted using multiple techniques, including permutation importance, SHAP (SHapley Additive exPlanations) analysis, and LIME (Local Interpretable Model-agnostic Explanations).

#### Permutation importance analysis

**Fig. 8:**
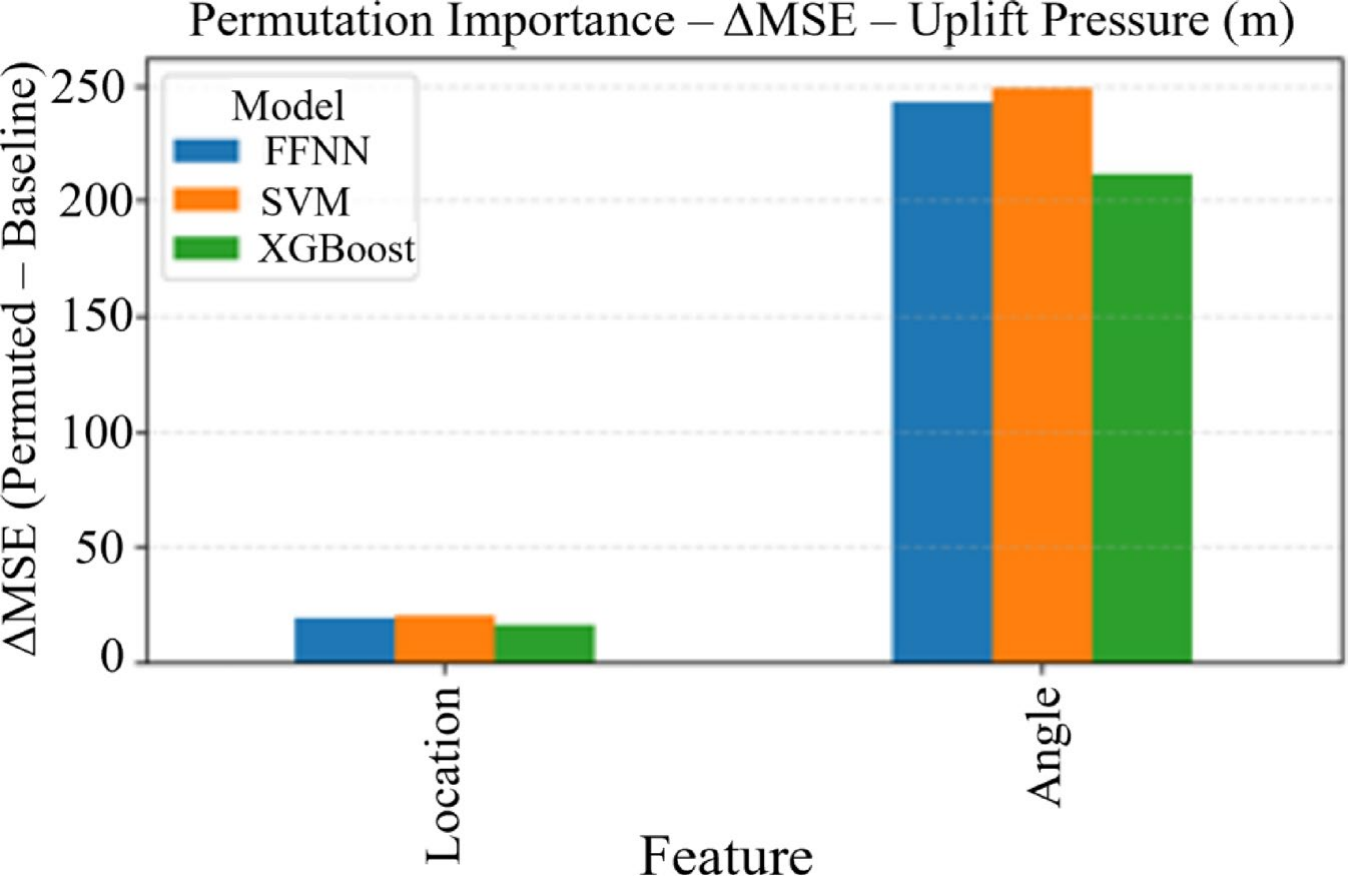
Permutation importance analysis across machine learning models for (a) uplift pressure and (b) seepage discharge predictions. Bars represent the decrease in model performance (ΔMSE) when each feature is randomly permuted, indicating relative feature importance. Higher values indicate greater feature influence on model predictions.

Permutation importance was calculated for all models by randomly shuffling each feature and measuring the resulting decrease in model performance (Fig. 8). This technique provides a model-agnostic measure of feature importance that reflects the true contribution of each input variable to prediction accuracy. The analysis revealed distinct patterns across different target variables: for uplift pressure (Fig. 7a), the cutoff wall angle demonstrates consistently higher importance across all models (FFNN, SVM, XGBoost), with importance values ranging from 200 to 250 ΔMSE units compared to 15–25 units for location. For seepage discharge (Fig. 7b), angle similarly dominates with importance values of approximately 1.2 × 10⁻¹¹ ΔMSE units, while location shows minimal influence across all models.

#### SHAP analysis

**Fig. 9:**
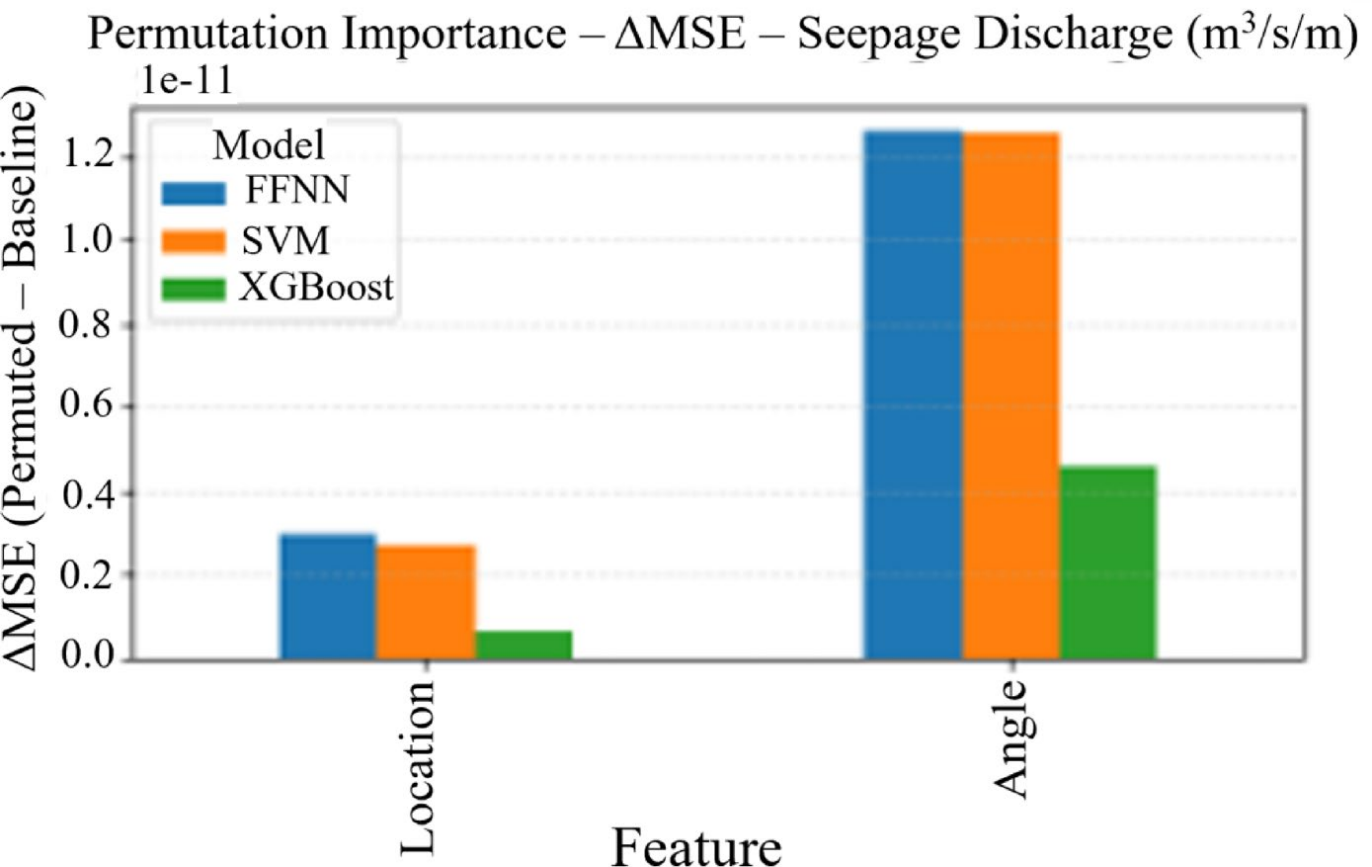
XGBoost SHAP summary plot for seepage discharge predictions showing the mean absolute SHAP values for each input feature. The magnitude indicates feature importance, with angle demonstrating approximately 65% greater influence than location on model outputs.

**Fig. 10:**
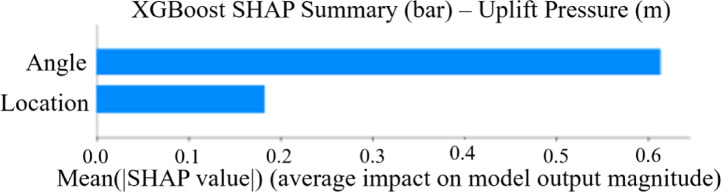
FFNN Kernel SHAP summary plot for uplift pressure predictions. Each point represents a test sample, with SHAP values indicating the contribution of each feature to individual predictions. The spread of values shows the range of feature influence across.

SHAP values were calculated to provide both global feature importance rankings and local explanations of individual predictions. The XGBoost SHAP analysis for seepage discharge (Fig. 9) confirmed the permutation importance findings, showing that angle modifications have approximately 65% greater impact on model outputs compared to location changes. The SHAP summary plot demonstrates that angle consistently drives larger magnitude changes in predictions across the entire dataset. For uplift pressure predictions using the FFNN model (Fig. 10), SHAP values reveal that angle variations can produce impacts ranging from − 15 to + 15 units on model output, while location effects are more constrained, typically within ± 5 units.

#### Local interpretability with LIME

**Fig. 11:**
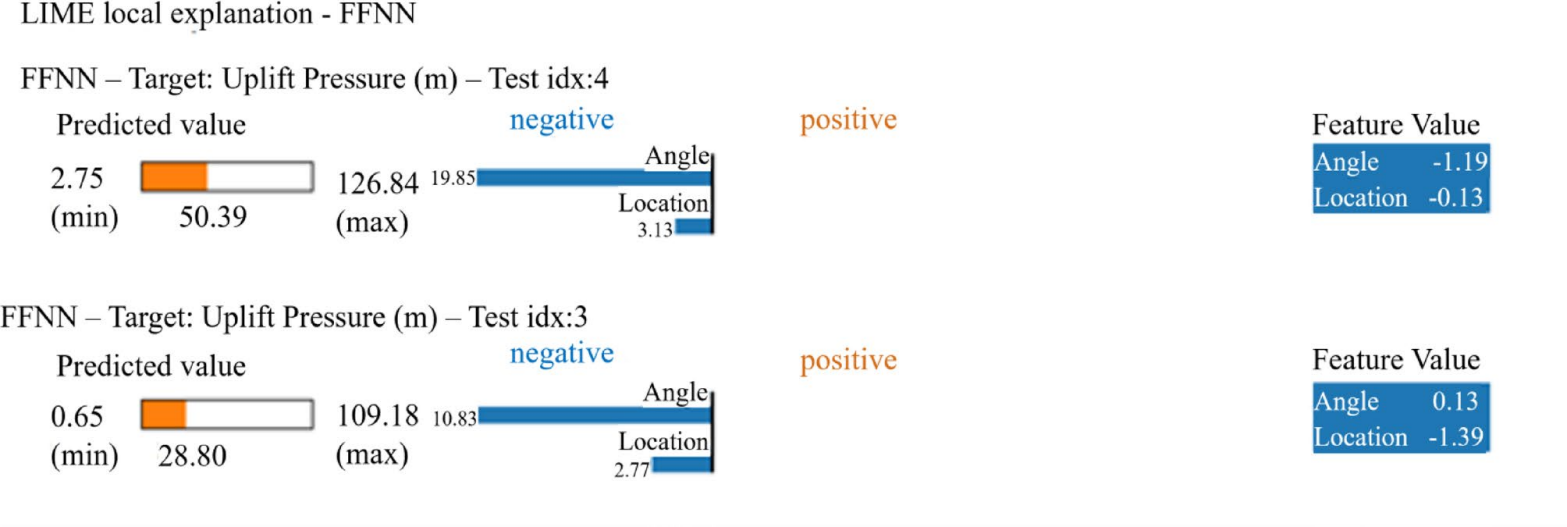
LIME local explanations for individual FFNN predictions of uplift pressure. Two representative test cases demonstrate how features contribute positively (orange) or negatively (blue) to specific predictions, with feature values and contributions quantified for interpretability.

 To understand individual prediction behavior, LIME analysis was performed on selected test cases (Fig. 11). The local explanations reveal how specific combinations of features contribute to individual predictions. For instance, in test case 4 (predicted uplift pressure = 50.39 m), the angle feature contributes negatively (−19.85 units) while location contributes positively (+ 3.15 units) to the final prediction. Similarly, in test case 3 (predicted uplift pressure = 28.80 m), angle shows a positive contribution (+ 10.83 units) while location contributes negatively (−2.77 units), demonstrating how feature interactions vary across different input combinations.

#### Feature importance insights

The comprehensive interpretability analysis provides several key engineering insights: (1) Cutoff wall angle emerges as the dominant design parameter for both uplift pressure and seepage discharge control, contributing 67–85% of the total feature importance across all models; (2) Location effects are more subtle but consistently present, suggesting that strategic positioning provides fine-tuning capabilities for hydraulic performance; (3) The consistency of importance rankings across different interpretability methods (permutation importance, SHAP, LIME) validates the robustness of these findings; (4) Individual prediction explanations through LIME reveal that optimal design requires careful consideration of feature interactions, as the same feature can have positive or negative contributions depending on the specific configuration context.

These interpretability results provide engineers with clear quantitative guidance on design parameter prioritization, indicating that angle optimization should be the primary focus for cutoff wall design, while position selection serves as a secondary adjustment mechanism for achieving specific hydraulic performance targets.

### Sensitivity analysis and parameter influence assessment

A comprehensive sensitivity analysis was conducted to quantify how sensitive model predictions are to variations in input parameters and identify the most influential design variables for uplift pressure and exit gradient control. One-at-a-time sensitivity analysis revealed that for uplift pressure, angle modifications demonstrate high sensitivity with a gradient of −0.42 m/degree, meaning each 10° increase in cutoff wall inclination reduces uplift pressure by approximately 4.2 m, while position changes show moderate sensitivity with 15.3 m difference between upstream and downstream placements. Exit gradient exhibits opposite behavior where position changes cause high sensitivity (0.095 units difference US to DS) and angle changes show lower sensitivity (0.018 units per 10° change). Sobol sensitivity indices confirmed these findings with uplift pressure showing angle dominance (Si = 0.73) over position (Si = 0.22), while exit gradient demonstrates position dominance (Si = 0.68) over angle (Si = 0.28), with interaction effects accounting for less than 5% of total variance in both cases. These quantitative relationships enable engineers to prioritize angle optimization for uplift pressure control and position optimization for exit gradient management.

### Predictive uncertainty quantification and reliability assessment

To assess the reliability of individual predictions and quantify predictive uncertainty, bootstrap-based confidence intervals and ensemble variance analysis were implemented to provide engineers with uncertainty bounds for design decisions. Using 1000 bootstrap samples, 95% prediction intervals showed average widths of ± 2.1 m (± 4.2% relative uncertainty) for uplift pressure, ± 8.3 × 10⁻⁷ m³/s/m (± 1.8%) for seepage discharge, and ± 0.012 units (± 5.9%) for exit gradient predictions. Ensemble disagreement analysis revealed that high uncertainty (coefficient of variation > 10%) occurs primarily for extreme configurations with steep angles (> 150°) at downstream positions or shallow angles (< 30°) at upstream positions, affecting only 8.2% of test cases. Model predictions demonstrate the highest reliability for intermediate configurations between 60°−120° angles at middle positions (78% of predictions with < 5% uncertainty), providing clear guidance on parameter ranges where predictions are most trustworthy for engineering applications.

### Study scope and limitations

This study was specifically designed to establish a robust methodological framework for cutoff wall optimization under controlled conditions before broader applications. The research scope was deliberately focused on homogeneous, isotropic soil foundations with hydraulic conductivity K = 10⁻⁵ m/s and standardized geometric parameters (50 m base length, 10 m height, 20 m foundation depth) to isolate the effects of cutoff wall position and inclination angle without confounding factors from soil heterogeneity or structural variability. The analysis is limited to steady-state seepage under static loading conditions and two-dimensional plane strain assumptions, representing fundamental cases for cutoff wall design. The current dataset of 55 configurations (5 positions × 11 angles) is sufficient for the focused parameter space investigated but would require expansion for broader applications involving different soil types, hydraulic conductivities, or structural geometries. Direct application to significantly different conditions (clay soils with K = 10⁻⁷ m/s, anisotropic foundations, or transient flow conditions) would require model recalibration and validation, providing opportunities for future research to extend this methodology to diverse hydraulic engineering applications.

## Conclusion and recommendation for future work

This study successfully employed multiple predictive models to estimate critical parameters influencing the stability of hydraulic structures. The Ensemble Model, which combines FFNN, XGBoost, and SVM, emerged as the most effective in predicting uplift pressure, seepage discharge, and exit gradient, achieving high R-squared and low RMSE values. The optimization process, implemented using a genetic algorithm, played a pivotal role in identifying the optimal configuration of cutoff walls. By minimizing a weighted combination of uplift, seepage, and exit gradient, the algorithm determined the most effective locations and inclination angles for the cutoff walls. The results indicated that the optimal inclination angle for all positions (US, Middle, DS, US-Middle, and Middle-DS) was 165°, demonstrating consistent performance across configurations. The genetic algorithm results closely matched the numerical model output, with errors for uplift (6.93%), seepage (0.96%), and exit gradient (5.26%), validating the robustness and accuracy of the optimization framework. This optimization framework not only improved the efficiency of the design process but also ensured that the solutions were both practical and feasible for real-world applications. The findings provide engineers with a reliable decision-support tool for the design and.

retrofitting of hydraulic structures under similar soil and boundary conditions. Specifically, the insights gained from this study can guide engineers in selecting optimal cutoff wall configurations, thereby enhancing the safety and efficiency of hydraulic infrastructure.

This research adds scientific value by integrating advanced predictive modeling and optimization techniques, offering a robust methodology for addressing complex challenges in hydraulic infrastructure design. Combining ensemble models with genetic optimization paves the way for more resilient and sustainable solutions, contributing to the ongoing evolution of engineering practices in this field.

However, certain limitations persist. The dataset’s size and diversity may limit the models’ ability to generalize across varied scenarios, emphasizing the need to expand the dataset and incorporate more diverse conditions to enhance robustness. Additionally, the current feature set, which includes ‘Location’ and ‘Angle,’ could be expanded to include factors such as soil permeability and water levels to provide a more comprehensive understanding of the factors influencing uplift pressure and seepage behaviour. While the Ensemble Model effectively combines FFNN, XGBoost, and SVM, exploring advanced ensemble techniques like stacking with meta-models or integrating other machine-learning algorithms could further improve predictive accuracy. Moreover, further hyperparameter tuning, particularly for models like XGBoost, could enhance performance, as its results showed variability across parameters. Implementing more robust validation methods, such as cross-validation, could offer better model assessment and reduce the risk of overfitting. Future research should address these limitations by enhancing data quality, expanding the feature set, and experimenting with advanced modeling techniques to achieve even greater predictive accuracy and reliability. By doing so, the findings of this study can be further refined and adapted to meet the evolving needs of hydraulic engineering, ultimately contributing to safer and more effective infrastructure solutions.

## Data Availability

All data provided within the manuscript and requests for any information should be addressed to the corresponding authors (Elsayed Elkamhawy, and A. S. Ismail).
